# Analysis of anti-*Plasmodium* IgG profiles among Fulani nomadic pastoralists in northern Senegal to assess malaria exposure

**DOI:** 10.1186/s12936-020-3114-2

**Published:** 2020-01-13

**Authors:** Mame Cheikh Seck, Julie Thwing, Aida Sadikh Badiane, Eric Rogier, Fatou Ba Fall, Pape Ibrahima Ndiaye, Khadim Diongue, Moustapha Mbow, Mouhamadou Ndiaye, Mamadou Alpha Diallo, Jules François Gomis, Aminata Mbaye, Tolla Ndiaye, Aminata Gaye, Mohamad Sy, Awa Bineta Déme, Yaye Die Ndiaye, Daouda Ndiaye

**Affiliations:** 1 Department of Parasitology, Faculty of Medicine and Pharmacy, Cheikh Anta Diop University, Dakar, Senegal; 2 Laboratory of Parasitology, Aristide Le Dantec Teaching Hospital, Dakar, Senegal; 3 Malaria Branch, Division of Parasitic Diseases and Malaria, Centers for Disease Control and Prevention, Atlanta, GA 30029 USA; 4 Senegal National Malaria Control Programme, Dakar, Senegal; 5 Department of Immunology, Faculty of Medicine and Pharmacy, Cheikh Anta Diop University, Dakar, Senegal

**Keywords:** Malaria, MSP-1_19_, CSP, LSA-1, Antibodies, *Plasmodium*, Fulani, Senegal

## Abstract

**Background:**

Northern Senegal is a zone of very low malaria transmission, with an annual incidence of < 5/1000 inhabitants. This area, where the Senegal National Malaria Control Programme has initiated elimination activities, hosts Fulani, nomadic, pastoralists that spend the dry season in the south where malaria incidence is higher (150–450/1000 inhabitants) and return to the north with the first rains. Previous research demonstrated parasite prevalence of < 1% in this Fulani population upon return from the south, similar to that documented in the north in cross-sectional surveys.

**Methods:**

A modified snowball sampling survey of nomadic pastoralists was conducted in five districts in northern Senegal during September and October 2014. Demographic information and dried blood spots were collected. Multiplex bead-based assays were used to assess antibody responses to merozoite surface protein (MSP-1_19_) antigen of the four primary *Plasmodium* species, as well as circumsporozoite protein (CSP) and liver stage antigen (LSA-1) of *Plasmodium falciparum*.

**Results:**

In the five study districts, 1472 individuals were enrolled, with a median age of 22 years (range 1 to 80 years). Thirty-two percent of subjects were under 14 years and 57% were male. The overall seroprevalence of *P. falciparum* MSP-1_19_, CSP and LSA-1 antibodies were 45, 12 and 5%, respectively. *Plasmodium falciparum* MSP-1_19_ antibody responses increased significantly with age in all study areas, and were significantly higher among males. The highest seroprevalence to *P. falciparum* antigens was observed in the Kanel district (63%) and the lowest observed in Podor (28%). Low seroprevalence was observed for non-*falciparum* species in all the study sites: 0.4, 0.7 and 1.8%, respectively, for *Plasmodium ovale*, *Plasmodium vivax* and *Plasmodium malariae* MSP-1. Antibody responses to *P. vivax* were observed in all study sites except Kanel.

**Conclusion:**

Prevalence of *P. falciparum* MSP-1_19_ antibodies and increases by study participant age provided data for low levels of exposure among this transient nomadic population. In addition, antibody responses to *P. falciparum* short half-life markers (CSP and LSA-1) and non-*falciparum* species were low. Further investigations are needed to understand the exposure of the Fulani population to *P. vivax*.

## Background

Malaria continues to cause significant morbidity and mortality, and according to the World Health Organization (WHO), an estimated 219 million malaria cases and 435,000 malaria deaths occurred worldwide in 2017 [1]. In Senegal, malaria transmission seasons are associated with rainfall, and transmission generally occurs from July to November, during the rainy season and at the beginning of the dry season. Malaria burden varies greatly from the semi-desert north (reported annual incidence < 5/1000 inhabitants) to the forested savannah in the southeast (reported annual incidence 250–450/1000 inhabitants) [2].

During the last 15 years, the Senegal National Malaria Control Programme (NMCP) has dramatically increased coverage of malaria prevention and case management activities nationwide, leading to a reduction of malaria prevalence from 6% in 2008 to < 1% in 2017 [3, 4]. Senegal has conducted four rounds of universal coverage distribution of long-lasting insecticide-treated nets (LLINs): 2010–2012, 2013–2014, 2016, and 2019. Artemisinin-based combination therapy (ACT) was introduced in 2006, rapid diagnostic tests (RDTs) were introduced in 2007, and were both widely available at both health facilities and the community level (in health huts and by community health workers performing home-based management) by 2010.

As a result, the burden of malaria has decreased throughout the country. In the north, many health facilities now report an annual incidence < 1 case per 1000 inhabitants, and the majority of malaria cases are diagnosed among those with history of travel outside the district in the last month [2]. The NMCP has set an ambitious target to interrupt malaria transmission in the north by 2020 [5]. Like much of the Sahel, Senegal hosts a population of Fulani nomadic pastoralists who lead their herds to the south in search of pasture during the dry season, where malaria prevalence is higher, and move northward with the first rains to spend rainy season in the north. There is concern that mobile populations who move between areas of low and high transmission may contribute to ongoing transmission in pre-elimination areas by becoming infected in higher transmission zones and returning to low transmission zones with circulating parasites [6, 7]. Mobile populations may contribute to re-introduction of malaria in areas in which it had been eliminated [8, 9]. To eliminate malaria, mobile populations such as nomads and migrant workers who frequently move between areas of high and low malaria transmission need access to malaria prevention and case management.

A recent study among nomadic pastoralists in northern Senegal found low access to health messaging, mass distributions of insecticide-treated nets, and malaria case management, however, contrary to expectations, malaria prevalence was very low (0.5% by blood smear and 0.6% by PCR) despite poor access [10]. Senegalese nomadic pastoralists are primarily Fulani, an ethnic group documented to have elevated immune responses to malaria and higher antibody seroprevalence compared to similarly exposed sympatric ethnic groups in other countries, such as Burkina Faso [11] and Mali [12]. Information about the immune response would be useful in understanding current malaria risk and designing control interventions. For example, partial immunity against malaria can be developed following repeated exposure, which limits blood-stage parasitaemia and prevents symptomatic illness and severe complications [13]. Many studies have shown that MSP-1_19_ IgG antibody, which has a long half-life, reflects the cumulative exposure to malaria, and response to this antibody is associated with protection from clinical disease with *P. falciparum* [14–16]. Information about the degree of protective immunity would help in anticipating the risk of severe disease in persons who develop malaria infection. Serologic responses to pre-erythrocytic antigens (circumsporozoite protein, or CSP, and liver-stage antigen 1, or LSA-1) provide information about recent changes in malaria transmission intensity [17]. Responses to the blood-stage MSP-1_19_ antigens of non-*falciparum* species can provide information about the local importance of these non-dominant parasites.

Dried blood spots collected from the previously described study among Fulani nomadic pastoralists in Senegal [10] were analysed for this current report using a multiplex bead-based assay in order to better understand current and historic levels of malaria exposure in this population and to determine if antibody seroprevalence reflected the low burden of malaria found by blood smear and PCR.

## Methods

### Study sites

**Fig. 1:**
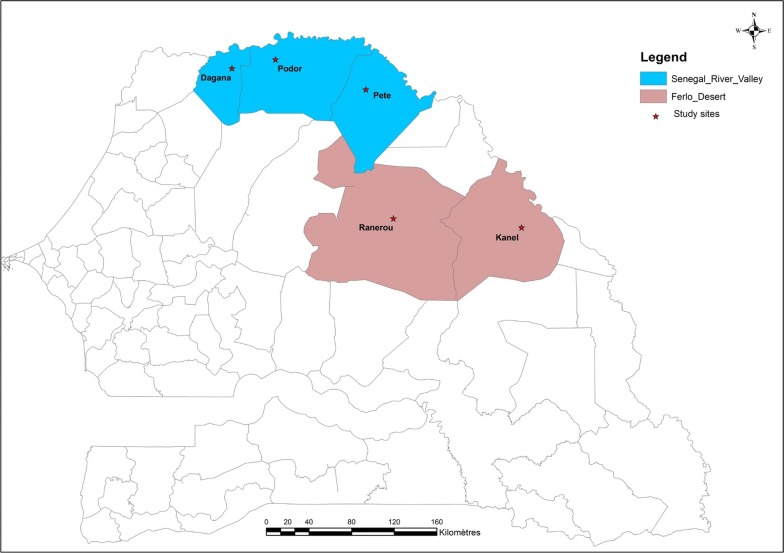
Map of study districts and sites in northern Senegal

The original, modified, respondent-driven sampling study was conducted in six districts in northern Senegal in the Senegal River valley and the Ferlo desert [10]. For this analysis, five of the six sites were used (in the excluded site, a large proportion of recruited nomadic pastoralists belonged to a different ethnic group). In these areas, the dry season is from November to June and the rainy season from July to October, and the main malaria vectors are *Anopheles gambiae* and *Anopheles arabiensis* [5]. The annual rainfall can reach 600 mm, increasing from north to south. Malaria transmission in these areas is highly seasonal, with transmission from July to December [18]. The Senegal River Valley stretches over 800 km from the coast, bordering Mauritania, to the border of Mali and along the Falémé tributary of the Senegal River. Three of the selected sites were in the Senegal River Valley, in the health districts of Dagana, Podor and Pete. The Ferlo desert is a vast Sahelian plain of over 75,000 sq km located along the Senegal River in northeastern Senegal; two of these selected sites were in the Ferlo desert, one in the health district of Ranerou and one in Kanel (Fig. 1).

### Study design, data and sample collection

Blood samples were obtained from September to October 2014 from Fulani nomadic pastoralists aged 6 months and older, who were returning to or had just returned to the north after staying in the south during the dry season. Consent/assent included permission to store samples for future testing not detailed in the original protocol. The details of the study design have been described elsewhere, including description of laboratory procedures [10]. Briefly, a respondent-driven survey with sampling modified for inclusion of minors was conducted. Respondent driven sampling is a variant of snowball sampling in which participants are asked to recruit a specified number of contacts and give details about the size of their social network, and uses a mathematical model to compensate for the non-random sampling. After obtaining informed consent, a questionnaire was administered for collecting socio-demographic data (age, gender and ethnicity) and ownership and use of insecticide-treated nets. For each participant, a rapid diagnostic test (RDT) and blood smear were performed. Any person with a positive RDT received the first line artemisinin-based combination therapy per national policy. Three to four drops of blood were collected on a Whatman 903 protein saver card (Sigma-Aldrich, St Louis, MO, USA) filter paper, dried at room temperature, sealed in plastic bags with silica gel and kept at room temperature until serological testing in June 2018.

### Laboratory methods

#### Serological assays

##### Antigens and peptides

Merozoite surface protein 19kD subunit (MSP-1_19_) recombinant antigens for *P. falciparum*, *P. vivax*, *P. malariae*, *P. ovale* were produced and purified by glutathione-*S*-transferase (GST) fusion protein as described previously [19]. A bead coupled to GST alone was also included in the multiplex panel to assess if any individuals in the study produced IgG against GST, but no persons were found to have anti-GST IgG as defined by an assay signal above 100 MFI-bg units. For *P. falciparum*, peptides for the circumsporozoite protein (CSP, (NANP) × 5 sequence) and liver stage antigen 1 (LSA-1) [20] were also included. Glutathione-*S*-transferase (GST) from *Schistosoma japonicum* was included as a non-binding control antigen.

##### Antigen coupling to beads

All antigens were coupled to magnetic beads (Luminex Corp, TX, USA) in the same manner as prior studies [21]. Briefly, beads were pulse vortexed, transferred to a microcentrifuge tube and centrifuged for 1.5 min at 13,000*g*. Supernatant was removed and beads were washed with 0.1 M sodium phosphate, pH 6.2 (NaP). Beads were activated by suspending in NaP with 5 mg/mL of EDC (1-ethyl-3-[3-dimethyl minutes propyl] carbodiimide hydrochloride) and 5 mg/mL sulfo-NHS (sulfo *N*-hydroxysulfosuccinimide) and incubated with rotation for 20 min at room temperature (RT) protected from light. After a wash with coupling buffer (50 mM 2-(4-morpholino)-ethane sulfonic acid, 0.85% NaCl at pH 5.0), antigens were coupled to beads in presence of coupling buffer for 2 h at an antigen concentration of 20 μg/mL for all MSP-1_19_ antigens, 60 μg/mL for LSA1, 30 μg/mL for CSP, and GST at 15 μg/mL. Beads were washed once with PBS, and suspended in PBS with 1% bovine serum albumin (BSA) with incubation for 30 min at RT by rotation. Beads were then resuspended in storage buffer (PBS, 1% BSA, 0.02% sodium azide and 0.05% Tween-20) and stored at 4 °C.

##### IgG antibody testing by bead-based assay

The bead-based multiplex technology was used. Briefly, a 6-mm circular punch was taken from the centre of each blood spot, corresponding to 10 μL of whole blood, for elution. Samples were diluted in 200 μL blocking Buffer B (PBS pH7.2, 0.5% Polyvinyl alcohol (Sigma), 0.8% Polyvinylpyrrolidine (Sigma), 0.1% casein (ThermoFisher), 0.5% BSA (Millipore), 0.3% Tween-20, 0.1% sodium azide, and 0.01% *Escherichia coli* extract to prevent non-specific binding, and stored at 4 °C until analysis. For the immunoassay, in 5 mL of reagent buffer (Buffer A: PBS, 0.5% BSA, 0.05% Tween-20, 0.02% NaN_3_), a bead mix was prepared with all regions included, and 50 μL bead mix was pipetted into each well of a BioPlex Pro plate (BioRad). Beads were washed 2 × with 100 μL wash buffer (PBS, 0.05% Tween 20), and 50 μL reagent mix [in 5 mL Buffer A: 1:500 biotinylated anti-human IgG (Southern Biotech), 1:625 biotinylated anti-human IgG_4_ (Southern Biotech), 1:200 streptavidin-PE (Invitrogen)] was added to all wells, then 50 µL samples (or controls) were added to the appropriate wells in singlet at a sample dilution of 1:50. Plates were incubated overnight with gentle shaking at RT and protected from light. The next morning (after ~ 16 h total incubation time), plates were washed 3×, and beads were re-suspended with 100 µL PBS and read on a MAGPIX machine (Luminex Corp, TX, USA). Median fluorescence intensity (MFI) assay signal was generated for a minimum of 50 beads/region. To give the final signal used for analyses, MFI from wells incubated with Buffer B alone was subtracted from each samples’ MFI to give a final value of MFI minus background (MFI-bg).

### Statistical analysis

Data were analysed using SPSS Statistics version 17 for Windows (IBM Corp., Armonk, USA). After log-transforming MFI-bg values for the IgG data for each antigen, a two-compartment finite mixture model was applied to each antigen to determine means and standard deviation of the putative seronegative population for each antigen’s signal intensity data. The seropositivity threshold cut-off was defined as the lognormal mean of the lower distribution plus three standard deviations. Results of the finite mixture model for calculation of cut-offs are presented in Additional file 1. The mixture model provided a poor fitting to the PfMSP1 data and a high standard deviation (Additional file 1), so the cut-off for this antigen was determined by the point where the first and second distributions crossed each other. An individual sample was considered seropositive for IgG against a particular malaria antigen if the MFI-bg assay signal for that sample was above the seropositivity threshold for that antigen. Given the potential for cross-reactivity of anti-MSP-1_19_ antibodies among the different malaria species, for generating estimates for seropositivity to the non-*falciparum Plasmodium* MSP-1_19_ antigens, the MFI for the non-Pf MSP-1_19_ was required to be both greater than the cut-off generated by the finite mixture model and greater than the PfMSP-1_19_ MFI for that sample. Differences between groups were evaluated using the Chi-square test for dichotomous variables and the nonparametric Kruskal–Wallis test followed by post hoc test to analyse pairs of groups for continuous variables. A p-value < 0.05 was considered significant. A reversible catalytic model was fit to the seropositivity by age data for each antigen, and the estimates for the serological conversion rate (SCR) and serological reversion rate (SRR) per year were directly calculated from the likelihood model [20].

## Results

### Characteristics of the study participants

A total of 1472 individuals were enrolled from study sites in five districts: Podor, Dagana, Pete, Kanel, and Ranerou. The median age of the study cohort was 22 years (IQR: 12–37). Other characteristics of the study population are detailed in Table 1. Of note, the composition of the Kanel sample was an outlier: 66.1% (95% CI 60.4–71.5) of subjects were males 20 or more years of age, compared to 35.4% (95% CI 33.0–37.9) for the sample overall.

**Table 1:** Demographic characteristics, use of bed nets and prevalence of falciparum malaria among study participants

	Dagana n (%)	Kanel n (%)	Pete n (%)	Podor n (%)	Ranérou n (%)	Total n (%)
Gender						
Male	131 (44.6)	259 (87.8)	155 (52.7)	166 (56.1)	124 (42.3)	835 (56.7)
Female	163 (55.4)	36 (12.2)	139 (47.3)	130 (43.9)	169 (57.7)	637 (43.3)
Age group in years						
0 to 9	63 (21.5)	28 (9.5)	65 (22.1)	69 (23.3)	75 (25.6)	300 (20.4)
10 to 19	62 (21.2)	57 (19.3)	60 (20.4)	79 (26.7)	81 (27.6)	339 (23.0)
20 to 29	52 (17.7)	85 (28.8)	45 (15.3)	42 (14.2)	65 (22.2)	289 (19.6)
30 to 39	43 (14.7)	48 (16.3)	42 (14.3)	42 (14.2)	34 (11.6)	209 (14.2)
≥ 40	73 (24.9)	77 (26.1)	82 (27.9)	64 (21.6)	38 (13.0)	334 (22.7)
Reported bed net use previous night [10]	21 (7.2)	192 (65.1)	242 (82.3)	224 (75.7)	215 (73.4)	894 (60.8)
Parasite prevalence by microscopy [10]	0 (0)	2 (0.7)	3 (1.0)	0 (0)	3 (1.0)	8 (0.5)
Parasite prevalence by PCR [10]	0 (0)	3 (1.0)	3 (1.0)	0 (0)	3 (1.0)	9 (0.6)

### Antibody responses against *Plasmodium falciparum* MSP-1_19_ antigen

**Fig. 2:**
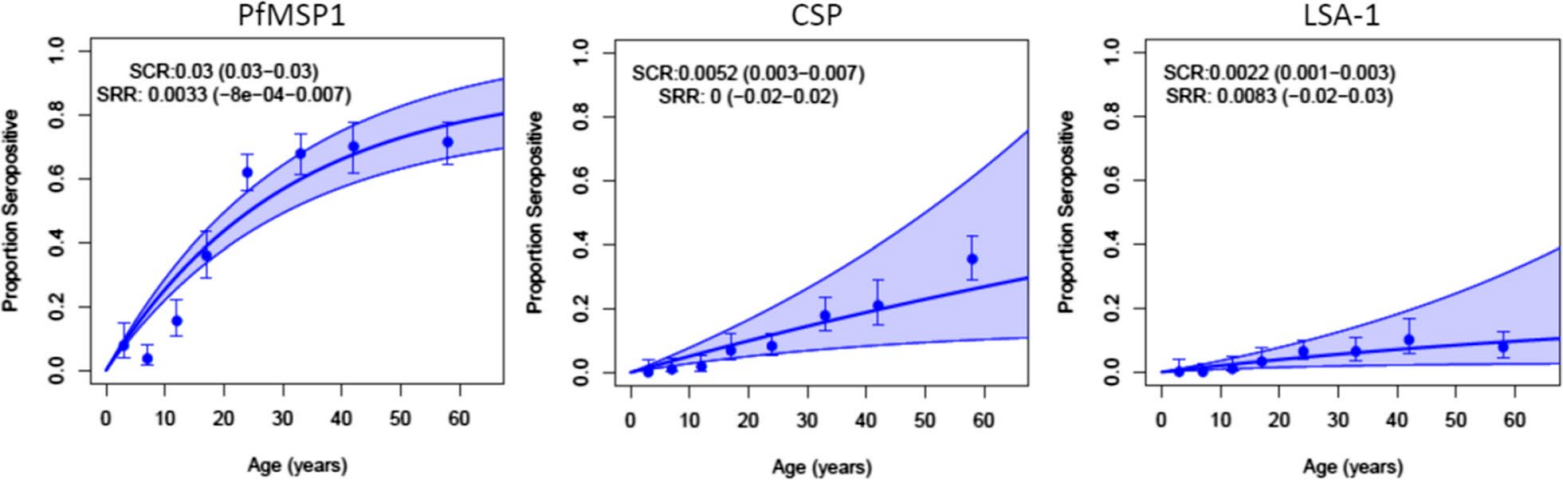
Seroprevalence curves for PfMSP1_19_, PfCSP, and PfLSA-1 by age-group

The overall seroprevalence of PfMSP-1_19_ was 45.0% (95% CI 42.5–47.7). The distribution of antibody response by district showed the highest seroprevalence to PfMSP-1_19_ in Kanel (63.4%, 95% CI 57.6–68.9) and Pete (55.8%, 95% CI 49.8–61.6), followed by Ranerou (39.9%, 95% CI 34.3–45.8), Dagana (37.9%, 95% CI 32.3–43.7), and Podor (28.0%, 95% CI 23.0–33.5). The highest estimated serological conversion rates (eSCR) for PfMSP-1_19_ were observed in Kanel and Pete, and lowest in Dagana and Podor (Table 2). By age group, the highest seroprevalence was found among participants aged over 40 years with 71.0% (95% CI 65.8–75.8), followed by age groups 30–39 years (67.9%, 95% CI 61.2–74.2), 20–29 years (61.9%, 95% CI 56.3–67.6), 10–19 years (26.0%, 95% CI 21.4–31.0), and decreasing to 5.3% among 0–9 years (95% CI 3.1–8.5) (Table 3), and seroprevalence curves demonstrate higher seroprevalence among those over the age of 20 years (Table 3, Fig. 2). Children under 10 years were far less likely to be seropositive for PfMSP-1_19_ than those 10 years or older (5.3%, 95% CI 3.1–8.5 vs 55.2%, 95% CI 52.5–58.0). Overall, positive antibody response was higher in males (51.7%, 95% CI 48.1–55.0) compared to females (36.4%, 95% CI 32.7–40.3).

**Table 2:** Seropositivity and estimated serological conversion rate (eSCR) for PfMSP-1_19_, CSP, and LSA-1 antibodies

	Dagana	Kanel	Pete	Podor	Ranerou
PfMSP-1_19_					
Seropositivity % (95% CI)	37.9 (32.3–43.7)	63.4 (57.6–68.9)	55.8 (49.9–61.6)	28.0 (23.0–33.5)	39.9 (34.3–45.8)
eSCR	0.022 (0.008–0.04)	0.063 (0.04–0.08)	0.047 (0.03–0.06)	0.015 (0.008–0.02)	0.029 (0.02–0.04)
CSP					
Seropositivity % (95% CI)	9.9 (6.7–13.9)	14.6 (10.8–19.1)	20.4 (16.0–25.5)	10.1 (6.9–14.2)	5.1 (2.9–8.3)
eSCR	0.0040 (0.004–0.008)	0.0055 (0.002–0.009)	0.0093 (0.002–0.02)	0.0045 (0.0004–0.008)	0.0026 (0.006–0.01)
LSA-1					
Seropositivity % (95% CI)	3.8 (1.9–6.6)	6.4 (3.9–9.9)	8.8 (5.9–12.7)	2.7 (1.2–5.3)	1.4 (0.4–3.5)
eSCR	0.0015 (0.0004–0.004)	0.0014 (0.0004–0.003)	0.0035 (0.0004–0.007)	0.0064 (0.002–0.01)	0.0013 (0.002–0.004)

**Table 3:** Antibody seroprevalence to *Plasmodium falciparum* antigens by age group

Antibody % (95% CI)	0–9 years	10–19 years	20–29 years	30–39 years	≥ 40 years
PfMSP-1_19_	5.3 (3.1–8.5)	26.0 (21.4–31.0)	61.9 (56.3–67.6)	67.9 (61.2–74.2)	71.0 (65.8–75.8)
CSP	0.7 (0.1–2.4)	4.4 (0.5–7.2)	8.3 (5.4–12.1)	17.7 (12.8–23.6)	29.6 (24.8–34.9)
LSA-1	0.0 (0.0–1.2)	2.4 (1.0–4.6)	6.2 (3.7–9.7)	6.2 (3.4–10.4)	8.7 (5.9–12.3)

### *Plasmodium falciparum* CSP antibody

Median MFI of CSP antibody production and the overall seroprevalence were 5.0 MFI units and 12.0% (95% CI 10.4–13.8), respectively. At 20.4%, seroprevalence was highest in Pete, as was the eSCR (Table 2**)**. Overall, CSP antibody responses increased significantly with age from 0.7% in 0–9 year olds to 29.6% among those 40 years and older (95% CI 24.8–34.9) (Table 3). Seroprevalence was higher among males (15.6%) than females (7.4%) (p < 0.0001).

### *Plasmodium falciparum* LSA-1 antibody

Median production of LSA-1 antibody was 3.0 MFI units and the overall seroprevalence was 4.6% (95% CI 3.6–5.8). No antibody response was observed in children less than 10 years old and seroprevalence among older children and adults increased significantly with age (Table 3). The highest seroprevalence was noted in Pete (8.8%) and Kanel (6.4%) followed by Dagana (3.8%), Podor (2.7%) and Ranerou (1.4%) (Table 2). The distribution of antibody response by gender showed a higher prevalence in males (6.1%) than females (2.7%) (p = 0.0018).

### Antibody responses against *Plasmodium malariae*, *Plasmodium ovale* and *Plasmodium vivax* MSP-1_19_ antigens

For *P. malariae*, MSP-1_19_ positivity was 1.8% and was observed in every site. *Plasmodium ovale* MSP-1_19_ positivity was 0.4%, and was observed only in the three northernmost sites on the Senegal River (Dagana, Podor, Pete), which border Mauritania. Of the 10 individuals positive for *P. vivax* MSP-1_19_ (0.7%), all but one were found in Dagana, Podor, or Pete (Table 4). Due to low number and percentages of persons seropositive, no trend was noted by age for non-falciparum MSP-1_19_ antibodies (Table 5).

**Table 4:** Seroprevalence of non-falciparum MSP-1_19_ antibodies by district

Study sites	Dagana	Kanel	Pete	Podor	Ranerou	Total
Overall N (%)	293 (19.9)	295 (20.1)	294 (20.0)	296 (20.1)	293 (19.9)	1471 (100)
MSP-1_19_ antibodies N (%)						
*P. malariae*	7 (2.4)	8 (2.7)	3 (1.0)	4 (1.4)	4 (1.4)	26 (1.8)
*P. ovale*	2 (1.0)	0 (0.0)	1 (0.3)	2 (0.7)	0 (0.0)	6 (0.4)
*P. vivax*	5 (1.7)	0 (0.0)	2 (0.7)	2 (0.7)	1 (0.3)	10 (0.7)

**Table 5:** Seroprevalence of non-falciparum MSP-1_19_ antibodies by age group

Age group (years)	0–9	10–19	20–29	30–39	≥ 40
Overall N (%)	300 (20.3)	339 (23.1)	289 (19.6)	209 (14.2)	334 (22.7)
MSP-1_19_ antibodies N (%)					
*P. malariae*	3 (1.0)	4 (1.2)	9 (3.1)	2 (1.0)	8 (2.4)
*P. ovale*	1 (0.3)	2 (0.6)	1 (0.3)	1 (0.3)	1 (0.3)
*P. vivax*	5 (1.7)	3 (0.9)	2 (0.7)	0	0

## Discussion

This work was part of an investigation to assess the degree of access to malaria control interventions and the burden of malaria among nomadic pastoralists in northern Senegal, as there was concern that this population could potentially contribute to ongoing transmission in very low transmission districts. Previously published analysis demonstrated that while access of nomadic pastoralists to malaria control interventions was low compared to non-nomadic populations in Senegal, malaria prevalence was also low, at 0.6% by nested PCR [10]. Assessment of malaria burden by light microscopy and PCR demonstrated parasite prevalence similar to resident populations, and molecular barcodes found among infections in this population did not match barcodes collected elsewhere in Senegal, giving no evidence as to their origin [10]. This current report found that measured antibody response to *P. falciparum* MSP-1_19_ (an erythrocytic antigen), PfCSP and PfLSA-1 (pre-erythrocytic antigens) demonstrated low current levels of recent exposure to falciparum malaria in this population. In addition, very low seroprevalence (< 2%) to non-falciparum IgG antibodies also shows that exposure to these other human malarias is sparse in this population of nomadic persons. Both parasite prevalence and malaria serology suggest that this population in fact has low malaria burden and low exposure, despite spending dry season in a higher transmission zone.

In low-transmission areas, parasite prevalence estimation using light microscopy is limited by detection sensitivity [11]. Even using PCR, the proportion with active infection in northern Senegal is too low to identify differences among populations without enormous sample sizes. Serology may be a reliable and sensitive tool for assessing the impact of malaria control strategies on malaria burden and transmission [12–14]. MSP-119 and CSP antibody response have been previously demonstrated as relevant surrogate markers for malaria transmission intensity in areas of low transmission [15–17]. In the setting of rapid scale-up of malaria control interventions in Senegal over the last decade, parasite prevalence decreased dramatically, and the incidence of malaria had fallen to less than 5 cases per 1000 people in the north by 2014 and less than 1 case per 1000 in some sites. The longevity of the antibody response toward blood-stage antigens (MSP-1_19_) allows an assessment of malaria over time, rather than a snapshot in time. Seroprevalence rates are thus higher than parasite prevalence and provide greater discrimination that can be used to provide a finer measure of malaria transmission than parasite prevalence.

The highest *P. falciparum* MSP-1_19_ seroprevalence rates were found in Kanel (63.3%), Pete (55.8%) and Ranérou (39.9%), the three districts in which malaria infections were detected. These districts also have higher reported annual incidence of confirmed malaria than the other two districts, Dagana and Podor, two of the northernmost districts in Senegal [18]. However, PfMSP-1_19_ seropositivity is known to increase with age [19–21], and positivity was dramatically lower among children under 10 years than among those 10 years and older, suggesting that those under 10 years have had less exposure to *P. falciparum* in the context of recent declines in transmission.

At two sites in the south and central regions of Senegal, where nomadic pastoralists spend the dry season, PfMSP-1_19_ seroprevalence rates (obtained by ELISA) of 53.1 and 31.5%, were reported in Velingara (south) and Keur Socé (central), respectively, among children under 10 years in 2010 [19]. While results obtained by ELISA cannot be directly compared to results of multiplex bead-based assays, the findings suggest substantially lower malaria exposure among nomadic pastoralist children under 10 years than among resident populations in the south and central regions of the country.

Low susceptibility to malaria among Fulani compared to sympatric populations has been documented in Burkina Faso [22] and Mali [23]. In other countries, Fulani populations have demonstrated higher levels of malaria antibody than sympatric populations with similar malaria exposure [24–26] and the relationship between high level of MSP-1_19_ antibody and protection from clinical malaria has been reported [27–32]. In the present cohort, antibody responses increased significantly with age in all study areas, which is in agreement with increasing immunity over time and seen in other studies in sub-Saharan Africa, Oceania and South America [19–21]. Regarding gender, the prevalence of antibody response was significantly higher among males except in Ranérou, and to a lesser extent in Dagana, where higher prevalence was observed in females. This might be due to differences in exposure to malaria infection between males and females; higher rates in some sites may reflect outdoor nocturnal activities, but these activities are typical for males, and the explanation for higher prevalence among females in some sites is not clear.

Seroprevalence to the pre-erythrocytic antigens PfCSP and PfLSA-1 was very low in this study, indicating the relative rarity of recent infections in the study population. These antibodies would most commonly be detected among individuals with frequent or recent exposure [33, 34]. Unlike merozoites, the sporozoite stages of *P. falciparum* are exposed to the immune system for only short periods after inoculation. Antibodies to PfCSP and PfLSA-1 were rare among children under 10 years, and somewhat more common among those 10 years and older, with relationship between seroprevalence of PfCSP and PfLSA-1 antibodies and age similar in all study sites. The PfCSP and PfLSA-1 seroprevalence by study site follows the same trends as PfMSP-1, with levels in Kanel and Pete higher than the other three sites.

Very low seroprevalence to the non-falciparum MSP-1_19_ antibodies was noted in all study sites, with carriage of anti-*P. malariae* IgG slightly more prevalent than *P. ovale* and *P. vivax*. While individuals seropositive for *P. malariae* MSP-1_19_ were found in all study sites, all the individuals seropositive for *P. ovale* MSP-1_19_, and 9 of the 10 persons seropositive for *P. vivax* MSP-1_19_ were from the Podor, Dagana, and Pete sites along the Senegal River, bordering Mauritania, a country where *P. vivax* and *P. ovale* have both been reported [35, 36].

*Plasmodium vivax* has traditionally been considered virtually absent from Western and Central Africa, due to the absence of the Duffy blood group, a blood receptor hitherto considered indispensable for the invasiveness of *P. vivax* into red blood cells, as more than 90% of the population in this area are believed to be Duffy negative [37]. However, the results suggest the presence of *P. vivax*, albeit at low levels, consistent with other recent reports suggesting the circulation of *P. vivax* in sub-Saharan Africa [38, 39]. In Mali, where *P. vivax* was considered absent, recent studies have indicated its presence in the country [40]. A recent study showed an unexpectedly high seroprevalence of *P. vivax* in a small sample of asymptomatic children from Kédougou in the southeastern Senegal, with a seroprevalence of 58% by ELISA [41]. Additional investigations are needed to provide clearer information, as the presence of *P. vivax*, especially in the northern areas where the NMCP is conducting pre-elimination strategies for malaria, would require diagnostic tools capable of detecting non-falciparum malaria and mixed infections, as well as updated treatment recommendations for non-falciparum malaria.

This study was not designed to compare nomadic pastoralists to surrounding populations, thus there are no available comparators among non-nomadic peoples in space and time. However, available evidence suggests that antibody response among nomadic pastoralists is more similar to resident populations in the north than to southern populations. While the majority of the sample of nomadic pastoralists are at least 15 years old, adult resident populations have not been sampled to permit comparison. While efforts were made to recruit a representative sample of nomadic pastoralists, due to their mobility, generating a sampling frame was not possible and a modified snowball approach was adopted. As this is a transient population, the differences between the population demographics by district are difficult to interpret since malaria exposure for an individual could have occurred in that district, or when the person was travelling outside of the district into another part of Senegal.

## Conclusion

Antibody response to short- and long-term antigenic markers to *P. falciparum* among Fulani nomadic pastoralists in northern Senegal was consistent with low exposure to malaria in the population. Due to the low seroprevalence of short-term antigens, and the previously reported low PCR prevalence in this population, it is likely the pastoralists would not pose a substantial risk of malaria re-introduction if local transmission is interrupted. Antibodies against to *P. vivax, P. ovale*, and *P. malariae* were very low in the entire population, but were found in persons enrolled at nearly all study sites. As Senegal moves towards pre-elimination in the north of the country, further investigations are needed to understand the circulation of non-falciparum *Plasmodium.*

## Supplementary information

**Additional file 1.** Fixed methods model distribution of two compartments for calculation of cut off values.

## Data Availability

The datasets used and/or analysed during the current study are available from the corresponding author on reasonable request.

## Abbreviations

MSP-1_19_: merozoite surface protein 19 kD fragment
CSP: circumsporozoite protein
LSA-1: liver stage antigen one
WHO: World Health Organization
NMCP: National Malaria Control Programme
LLINs: long-lasting insecticide-treated nets
RDT: rapid diagnostic tests
MFI: median fluorescence intensity
NaP: sodium phosphate
EDC: 1-ethyl-3-[3-dimethyl minutes propyl] carbodiimide hydrochloride
sulfo-NHS: sulfo *N*-hydroxysulfosuccinimide
RT: room temperature
mM: millimol
NaCl: sodium chloride
BSA: bovine serum albumin
PBS: phosphate buffer saline
