# Supplementary material for: Analysis of anti-Plasmodium IgG profiles among Fulani nomadic pastoralists in northern Senegal to assess malaria exposure
Source: Malar J. 2020 Jan 13;19:15. doi: 10.1186/s12936-020-3114-2 (PMC6958760; doi:10.1186/s12936-020-3114-2)
Supplement: Supplementary file 1 — Additional file 1. Fixed methods model distribution of two compartments for calculation of cut off values. [file 12936_2020_3114_MOESM1_ESM.docx]

Additional file 1: Fixed methods model distribution of two compartments for calculation of cut off values

| 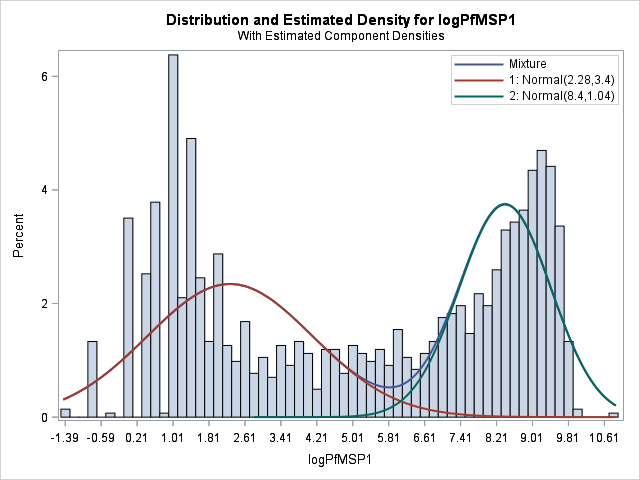 | 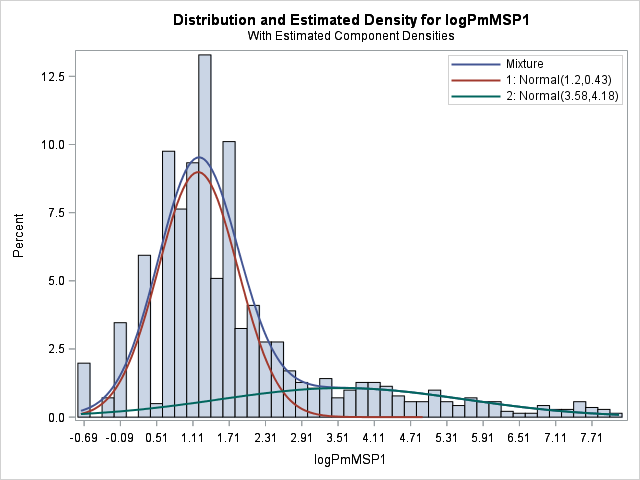 |
| --- | --- |
| 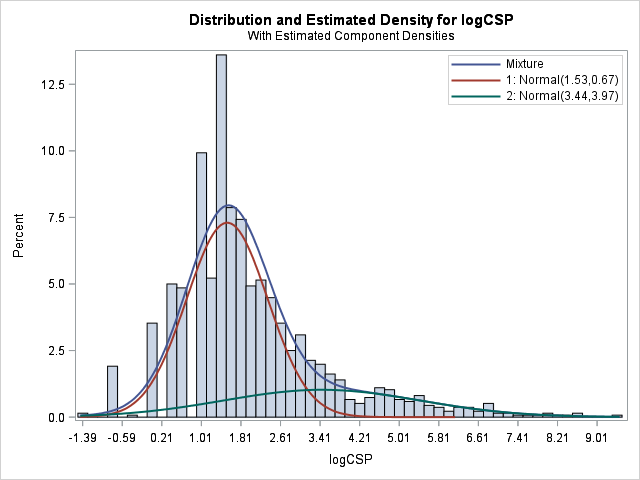 | 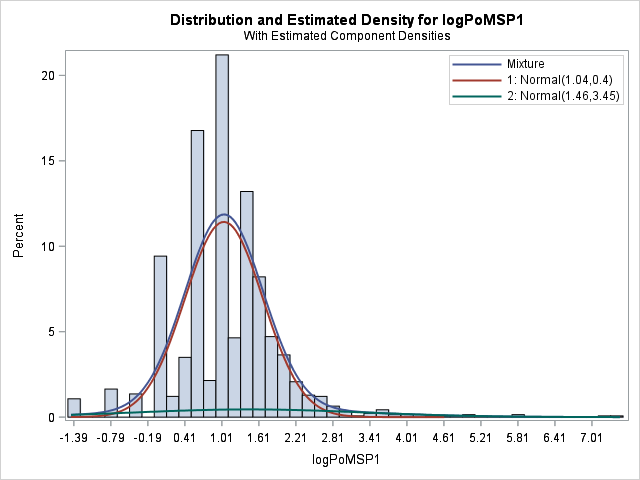 |
| 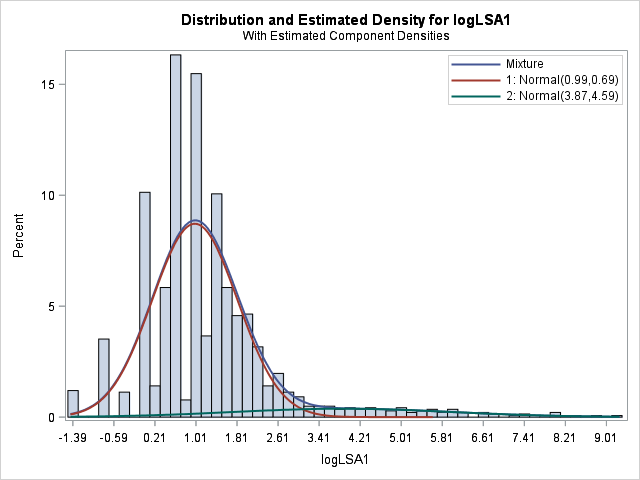 | 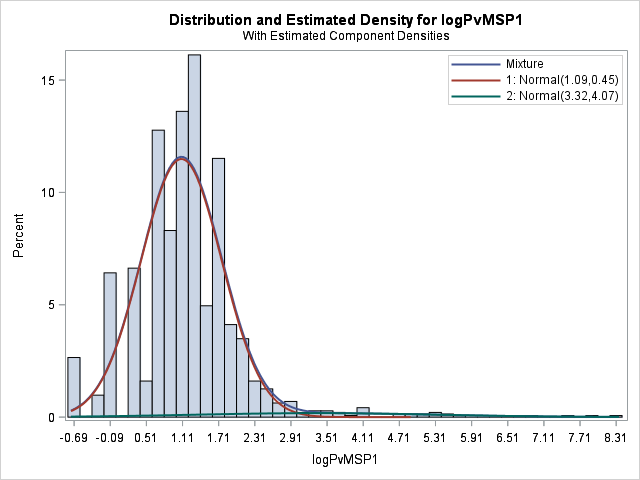 |

|  | Mean | Standard deviation | Mean + 3SD | Cut-off |
| --- | --- | --- | --- | --- |
| PfMSP1 | 2.28 | 1.84 | **6.2*** | **493** |
| PmMSP1 | 1.20 | 0.66 | 3.17 | **24** |
| PoMSP1 | 1.04 | 0.63 | 2.94 | **19** |
| PvMSP1 | 1.09 | 0.67 | 3.10 | **22** |
| LSA1 | 0.99 | 0.83 | 3.47 | **32** |
| CSP | 1.53 | 0.82 | 3.99 | **54** |

*Cut off determined visually

‘Mean’ is for log-transformed MFI-bg values

‘cutoff ‘ as exponentiated back to linear scale
